# Mutation analysis of the Gadd45 gene at exon 4 in atypical fibroxanthoma

**DOI:** 10.1186/1471-5945-9-1

**Published:** 2009-01-07

**Authors:** Akio Sakamoto, Shizuka Akieda, Yoshinao Oda, Yukihide Iwamoto, Masazumi Tsuneyoshi

**Affiliations:** 1 Department of Orthopaedic Surgery, Graduate School of Medical Sciences, Kyushu University, Fukuoka, Japan; 2 Department of Anatomic Pathology, Graduate School of Medical Sciences, Kyushu University, Fukuoka, Japan

## Abstract

**Background:**

Atypical fibroxanthoma (AFX) histologically mimics high-grade sarcoma in the skin, although it follows a benign clinical course. AFX occurs in the sun-exposed skin and for this reason, an association with ultraviolet light has long been suspected. Bax and Gadd45 are p53 effector proteins. Bax is a programmed cell death protein and belongs to the Bcl-2 family. Gadd45 is a multifunctional DNA damage-inducible gene associated with the process of DNA damage.

**Methods:**

Immunohistochemical expression of Bax was analyzed in 7 cases of AFX, and in 7 cases of benign fibrous histiocytoma (BFH) used as a comparison. The expression pattern of Bax was compared to previously reported p53 and Gadd45 expressions in a correspondent series. Mutation of the Gadd45 gene at exon 4 was also analyzed in AFX.

**Results:**

AFX and BFH showed immunoreactivities respectively for Bax (3/7, 0/7), Gadd45 (4/7, 1/7) and p53 (2/7, 0/7). There was no exact correlation between p53 expression and Bax or Gadd45 expression. However, the pattern of expression between Bax and Gadd45 was also the same, with the exception of one case. No mutation of the Gadd45 gene at exon 4 was observed in a series of 6 AFX cases where DNA was available (0/6).

**Conclusion:**

These results suggest a possible association between Bax and Gadd45 in AFX, and may refute any possibility of dysfunction of Gadd45 in terms of gene mutation, at least at exon 4 of the Gadd45 gene.

## Background

Atypical fibroxanthoma (AFX) is a nodular ulcerative lesion arising from the sun-exposed skin of the head and neck, typically in the elderly [1,2]. Solar elastosis associated with UV-radiation has been commonly observed in AFX cases [3]. Association between AFX and ultraviolet (UV) radiation has been suspected. On the other hand, in its less common forms with weakened association with UV, AFX occurs on the extremities and the trunk [1,2]. AFX is composed of spindle, plump, epithelioid and bizarre cells, in various proportions, arranged in haphazard, vaguely fascicular or storiform patterns. These histological features of AFX mimic those of high-grade sarcoma, such as malignant fibrous histiocytoma or leiomyosarcoma [2,4], which occurs deep within soft tissue.

The tumor-suppressor protein p53 is a transcriptional activator which is involved in cell-cycle control, DNA repair, apoptosis and chromosome/genome instability. UV-induced p53 gene mutations occurring at dipyrimidine sites have been demonstrated in AFX, suggesting a central role for UV radiation in the pathogenesis of AFX [5]. These p53 functions are mediated through its transcriptional target (effector) proteins, such as Bax and Gadd45 (growth arrest and DNA damage inducible) [6].

Bax is a pro-PCD (programmed cell death) protein and belongs to the Bcl-2 family [7]. Furthermore, Bax is important for p53-dependent PCD [8,9]. Gadd45 is a DNA damage-responsive gene that is rapidly induced by agents that cause DNA damage, including UV radiation. Gadd45 is a multifunctional protein which has roles to play in cell-cycle arrest, genomic stability, nucleotide excision repair, chromatin accessibility and apoptosis [10-12]. Although mutation of the Gadd45 gene is not common in tumors [13,14], some research has been reported showing that point mutations of Gadd45 gene at exon 4 were found in 13.6% of pancreatic cancer cases, suggesting the possibility that Gadd45 is dysfunctional in this tumor type [15]. We have previously reported the expression of Gadd45 in a series of AFX without mutation analysis [16].

In the current study, we evaluate the immunoexpression pattern of Bax in AFX. The existence of a mutation at exon 4 of the Gadd45 gene is also examined in order to refute the possibility of any dysfunction of Gadd45, regardless of its expression. We also discuss the correlation of immunoexpression among Bax, Gadd45 and p53 in the correspondent cases. We use benign fibrous histiocytoma (BHF), or dermatofibroma, as the comparison. BHF is a benign fibrohistiocytic tumor composed of a mixture of fibroblastic and histiocytic cells, most commonly seen in the dermis and superficial subcutis [2].

## Methods

### Cases of AFX and BFH

Seven cases of AFX and seven cases of BFH as a comparison were collected from the histopathological files at the Department of Anatomic Pathology, Kyushu University. The BFH cases were collected at random. These cases of AFX and BFH for Bax immunohistochemical analysis were exactly the same cases as those used for p53 immunohistochemical analysis in a previous report [17]. As for Gadd45 immunohistochemical analysis, although the cases were also the same as those used in our previous study [16], a few of those earlier cases were omitted from the current study, due to a lack of sufficient available materials. In the current study, an association among the expression of Bax, Gadd45 and p53 was compared in the correspondent cases. The research was performed under the approval of the Department of Anatomic Pathology and the Department of Orthopaedic Surgery, Kyushu University. Patients were informed that resected tumor samples might be used for research analysis. The explanation and the consensus were recorded on their medical charts. The research was performed ethically, in compliance with the Helsinki Declaration. In addition, the study was performed anonymously in order to protect the identity of the patients.

### Immunohistochemical staining

Four-micron-thick histological sections of 10% formalin-fixed and paraffin-embedded materials were deparaffinized. After dehydration, endogenous peroxidase was blocked by methanol containing 0.3% H_2_O_2 _for 30 min. The sections were incubated with the primary antibody at 4°C overnight, followed by reaction with the streptavidin-biotin complex method using an SAB-PO kit (Nichirei, Tokyo, Japan). The sections were then finally reacted in a 3,3'diaminobenzidine, peroxytrichloride substrate solution and counterstained with hematoxylin or methyl green. The antibody used in this study was gout polyclonal antibody for Bax. Its dilution was 1:200 for anti-Bax (P-19, Santa Cruz Biotechnology, Santa Cruz, CA). Specimens were pretreated by heating in a microwave oven. Immunoexpression results of Gadd45 (H165-X; Santa Cruz Biotechnology, Santa Cruz, CA, USA) [16] and p53 (PAb1801, Oncogene Research Products, San Diego, CA, USA) [17] have been reported previously.

### Formalin-fixed, paraffin-embedded tissue DNA extraction

DNA was extracted from a paraffin-embedded tissue section as follows. Paraffin was removed with xylene, and then the sample was washed twice with 100% ethanol and subsequently dried. The tissue was suspended in digestion buffer (100 mM sodium chloride, 10 mM Tris-hydrochloric acid, 25 mM EDTA, and 0.5% sodium dodecyl sulfate) containing 10 mg proteinase K, before being incubated overnight at 55°C. DNA, precipitated by adding twice the volume of ethanol, was washed with 70% ethanol, before being resuspended in TE buffer (10 mM Tris, 1 nM EDTA) for storage at 4°C.

### Polymerase chain reaction (PCR) and direct sequencing

A fragment of the Gadd45 gene at exon 4 was amplified by PCR. The PCR primers used were 5'-TTTGTTTCCAGAATCCACATTC-3' (sense) and 5'-AAAACTTCAGTGCAATTTGG-3' (antisense). The PCR was run in 35 cycles consisting of denaturation at 94°C for 1 min, annealing at 56°C for 45 sec, and extension at 72°C for 1 min. The amplified PCR fragments were purified by Purification System (BenevBio, Mission Viejo, CA). After the purification, direct sequencing was carried out by the dideoxy chain termination method using a Perkin Elmer ABI 3100 sequence analyzer (Applied Biosystems, Foster City, CA). Antisense primer was used as the sequencing primer.

### Assessment of immunoreactivity

Neoplastic cells that showed distinct nuclear or cytoplasmic staining, depending on the antibodies used, were considered positive. Positive cut-off values for each type of immunoexpression were adopted as follows; more than 10% for Bax in the nuclei [18], more than 25% for Gadd45 in the cytoplasm [19], and more than 5% for p53 in the nuclei [17].

### Statistical analysis

The data regarding immunohistochemistry were analyzed using Fisher's exact test. A *P *value of less than 0.05 was considered to indicate statistical significance.

## Results

### Clinical features

The clinical features are listed in Table 1. The average ages (in years) of patients with these lesions were as follows; AFX (n = 7; average age, 66.0; range, 46–84) and BFH (n = 7; average age, 29.6; range, 18–38). AFX showed a male-female predominance of 6 to 1. BFH occurred in 2 males and 5 females. AFX occurred on the sun-exposed skin of the scalp (1 case), face (1 case), auricle (2 cases) and finger (2 cases) in 6 cases, while it occurred on the leg in the other one case. BFH occurred at various sites of the trunk and the extremities (chest wall, 1 case; back, 1 case; thighs, 2 cases; forearm, 1 case; and legs, 2 cases) but none of these lesions occurred on sun-exposed parts. No recurrence was observed among these 7 AFX and 7 BFH cases.

**Table 1:** Clinical and immunohistochemical features of AFX and BFH

Lesion	Age/Sex/Location	Bax	Gadd45*	Gadd45 mutation at exon 4	p53*
A3 AFX	82/M/Scalp	-	-	NA	-
A7 AFX	84/M/Face	-	+	-	-
A10 AFX	65/M/Auricle	-	-	-	+
A14 AFX	71/M/Auricle	+	+	-	+
A16 AFX	50/M/Finger	-	-	-	-
A17 AFX	46/M/Finger	+	+	-	-
A19 AFX	64/F/Leg	+	+	-	-
					
B8 BFH	18/F/Thigh	-	-	NA	-
B9 BFH	38/F/Back	-	-	NA	-
B12 BFH	32/M/Thigh	-	-	NA	-
B13 BFH	35/F/Leg	-	+	NA	-
B16 BFH	30/F/Chest wall	-	-	NA	-
B17 BFH	23/M/Forearm	-	-	NA	-
B21 BFH	31/F/Leg	-	-	NA	-

AFX, atypical fibroxanthoma; BFH, benign fibrous histiocytoma (BFHs were selected at random from our histological files); *, data were reported previously; NA, not assessed.

### Histological features

**Figure 1:**
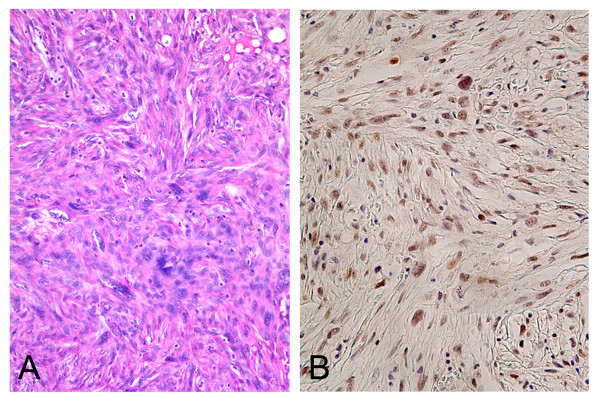
**Atypical fibroxanthoma demonstrates the proliferation of atypical spindle or polygonal cells arranged in fascicles, in a haphazard or disorderly pattern**. (A). Atypical fibroxanthoma shows positive nuclear immunoreaction for Bax (B). (H&E staining, A; × 180, Immunohistochemistry, B; × 200).

AFX is a nodular ulcerative lesion composed of various proportions of a mixture of spindle and pleomorphic cells, arranged in a haphazard or disorderly pattern (Figure 1). The BFH cases were located in the dermis or the superficial subcutis. BFH consisted of fibroblastic cells and histiocytic cells arranged in short interlacing fascicles or a vague storiform pattern. The tumor cells of BFH demonstrated no pleomorphism.

### Immunohistochemical findings

Immunohistochemical data are shown in Tables 1 and 2. The immunohistochemical data of Gadd45 [16] and p53 [17] in AFX and BFH have been reported previously. AFX showed positive expression of Bax in 3 cases (3/7; 43%), of Gadd45 in 4 cases (4/7; 57%) and of p53 in 3 cases (2/7; 29%) among a total of 7 cases. As for BFH, Bax (07; 0%), Gadd45 (1/7; 14%) and p53 (0/7; 0%) expressions were virtually absent in the 7 BFH cases, with the exception of 1 case that showed Gadd45 expression (1/7; 14%). The expression pattern of Bax and Gadd45 was also the same, with 3 cases positive for both and 3 cases negative for both, the one exception being one AFX case which was positive for Gadd45, but negative for Bax. Therefore, it seemed that there was a correlation in the expression between Bax and Gadd45 in the AFX cases. On the other hand, there seemed to be no correlation between p53 expression, and the expression of Bax or Gadd45. The frequency of Bax in AFX (3/7; 43%) was statistically higher than that in BFH (0/7; 0%) (p < 0.05), whereas there was no significant difference in Gadd45 and p53 expression between the AFX and BFH cases (p > 0.05).

**Table 2:** Immunohistochemical features of atypical fibroxanthoma and benign fibrous histiocytoma

Lesion	Bax	Gadd45*	Gadd45 mutation at exon 4	p53*
AFX	3/7 (43%)**	4/7 (57%)	0/6 (0%)	2/7 (29%)
BFH	0/7 (0%)**	1/7 (14%)	NA	0/7 (0%)

AFX, atypical fibroxanthoma; BFH, benign fibrous histiocytoma (BFHs were selected at random from our histological files); *, data were reported previously; **, p < 0.05; NA, not assessed.

### Mutation of Gadd45 at exon 4

Mutation of the Gadd45 gene at exon 4 was not observed in the series of 6 AFX cases where DNA was available (0/6; 0%), regardless of Gadd45 immunoexpression.

## Discussion

AFX typically occurs on the head and neck of sun-exposed skin. This fact has long suggested a role for sun exposure in the tumorigenesis of AFX. UV radiation from sunlight is an important risk factor for skin cancer [20]. UV-induced p53 gene mutations occurring at dipyrimidine sites have been demonstrated in AFX, suggesting a central role for UV radiation in the pathogenesis of AFX [5,17]. p53 protein is a key regulator for cell-cycle arrest and apoptosis induced by DNA damage [21]. Therefore Bax and Gadd45 are the central downstream effectors of p53 [6]. It has been reported that low doses of UV-radiation induced a rapid p53 accumulation followed by a decrease which was correlated with a transient cell-cycle withdrawal and presumably also DNA repair [22]. The association between decreased DNA repair ability and AFX may be supported by a report showing that patients with xeroderma pigmentosum had AFX, and it is known that xeroderma pigmentosum is related to DNA repair defects [23,24]. On the other hand, high and sustained levels of p53 were observed after a high dose of UV-radiation in cells undergoing apoptosis [22]. Therefore, regulation of p53 target genes was highly dependent upon the radiation dose used, with high doses inducing only Bax and Gadd45 expression [22]. In the current study, it seemed that there was a correlation between Bax and Gadd45 in these AFX cases. These associations of Bax and Gadd45 noted in AFX may be consistent with p53 effector upregulation against UV radiation, although there seemed to be no correlation between p53 expression, and the expression of Bax or Gadd45.

Bax expression has been reported to be associated with histological grade [25] and unfavorable prognosis [26] in ovarian tumors. In the current study, Bax was expressed in about 50% of the AFX cases. The frequency of Bax expression in AFX was significantly higher than that in BFH. This difference suggests the essential difference between the two lesions. Bax is a pro-PCD (programmed cell death) protein and belongs to the Bcl-2 family [7]. As far as we are concerned, there have been no reports presenting a direct comparison between AFX and BFH in terms of apoptotic behavior. However, it has been reported that apoptotic behavior in AFX is as high as that in high-grade sarcoma of MFH [27]. In addition, another research article has reported that there is a significant difference between MFH and BFH [28]. Therefore, it is reasonable to assume that the apoptotic behavior of AFX is higher than that of BFH. Moreover, the expression of Bax in AFX may reflect apoptotic behavior.

We have previously reported the expression of Gadd45 in a series of AFX, in which mutation of the Gadd45 gene was not analyzed [16]. Gadd45 gene mutation is uncommon in human tumors [13,14]. However, point mutations were found at exon 4 of the Gadd45 gene in 13.6% of pancreatic cancer cases, thereby suggesting that the possible dysfunction of Gadd45 plays a role in tumor development in certain types of tumors [15]. In the current study, no mutation of the Gadd45 gene at exon 4 was seen in a series of AFX, regardless of the immunohistochemical expression of Gadd45. This result may refute any possibility of dysfunction of Gadd45 in terms of gene mutation, at least at exon 4 of the Gadd45 gene.

AFX untypically occurs outside the head and neck region, such as in the extremities. The site-specific difference of AFX between the head and neck region and other regions is not well known. In the current series, 4 out of 7 cases occured in the head and neck region, while 3 cases occured in the extremities of the fingers and the leg. The expression of Gadd45 in AFX was more frequent than that in BFH, which was also true for the expression of Bax; BFH rarely demonstrated Gadd45 or Bax expression. Bax expression was seen in 1 out of 4 AFX cases in the head and neck region (25%), but in 2 out of 3 AFX cases (67%) in the extremities. On the other hand, Gadd45 expression was seen in 2 out of 4 in the head and neck region (50%), but in 2 out of 3 cases in the extremities (67%). It might be possible that the expression of Bax or Gadd45 in AFX is more prominent in the extremities, rather than in the head and neck region. Namely, the expression of Bax or Gadd45 in AFX may differ in a site-specific manner, possibly reflecting its pathogenesis. Further study will be needed in order to characterize the site-specific difference of AFX.

It remains controversial whether AFX and MFH are actually the same entity, or not. It seems that AFX and MFH share histological and immunological features. A histiocytic/macrophage marker, CD68, is positive in more than half of all AFX cases [29,30]. CD10 has been expressed in almost all cases of MFH, as well as in cases of AFX [16,31]. However, the number of AFX cases with moderate/strong or diffuse immunoreactivity for CD99 is significantly larger than that of MFH cases [32]. Reduced immunoexpression for CD74 in AFX may be characterized compared to that in MFH [33]. From a molecular aspect, AFX is characterized by a diploid pattern, while the majority of chromosomal changes in MFH are of an aneuploid pattern [34,35]. H-, K-, and N-ras gene mutations are not present in AFX, whereas MFH has H- and K-ras gene mutations, although only a small number of cases was studied [36]. In contrast to MFH, AFX is strongly associated with UV radiation in its pathogenesis. The formation of DNA photoproducts by UV radiation has been reported to be responsible for the development of skin cancer [37]. DNA photoproducts can interfere with the binding of several important cell-cycle regulatory and DNA damage-responsive transcription factors [37]. It has been reported that the accumulation of DNA photoproducts may play an important role in the pathogenesis of AFX [17]. It is also possible that AFX and MFH share the same pathway which determines their morphology, but that AFX and MFH may have different pathways of biological behavior which determine their clinical behavior.

## Conclusion

In the current study, we analyzed the immunohistochemical expressions of the p53 effector proteins of Bax and Gadd45 in AFX. Possible association between Bax and Gadd45 without gene mutation at exon 4, was observed. This result may refute any possibility of dysfunction of Gadd45 in terms of gene mutation, at least at exon 4 of the Gadd45 gene.

## Competing interests

The authors declare that they have no competing interests.

## Authors' contributions

**AS **drafted the manuscript. **AS **and **SA **carried out the mutation analysis. **YO **participated in the design of the study. **YI **and **MT **conceived of the study, and participated in its design and coordination and helped to draft the manuscript. All authors read and approved the final manuscript.

## Pre-publication history

The pre-publication history for this paper can be accessed here:


